# No evidence that experiment aversion is not a robust empirical phenomenon

**DOI:** 10.1073/pnas.2317514120

**Published:** 2023-12-04

**Authors:** Burcak Bas, Joachim Vosgerau, Rachele Ciulli

**Affiliations:** ^a^Department of Marketing, WU Vienna (Vienna University of Economics and Business), Vienna 1020, Austria; ^b^Department of Marketing, Bocconi University, Milan 20136, Italy; ^c^Marketing Department, The Wharton School, University of Pennsylvania, Philadelphia, PA 19104

Mazar et al. (1) conducted a direct replication and six conceptual replications of the studies demonstrating experiment aversion (2, 3), the tendency to rate an experiment as less favorable than its treatment arms. Based on their findings, Mazar et al. conclude that experiment aversion “is neither generalizable nor robust” (1). We are questioning whether Mazar et al.’s findings justify this conclusion.

First, in the direct replication, the authors perfectly replicate experiment aversion, testifying to the replicability of experiment aversion. In that sense, experiment aversion constitutes a robust empirical finding.

In the six conceptual replications testing the generalizability of experiment aversion, the authors changed the original scenarios in a number of ways. For example, the authors employed six rather than one dependent variable to measure evaluations of hospitals that conduct experiments and instantiate treatment arms, asked participants to evaluate several hospitals rather than the decision of one doctor, described the hospitals’ actions in the past rather than the present tense, etc. Unfortunately, these deviations from the original studies (2, 3) were not systematically and orthogonally manipulated, so it is not clear which changes contributed to not finding evidence of experiment aversion. Even more troubling, evidence for experiment aversion was found on some of the authors’ measures but not on others.

While we agree that these findings suggest that experiment aversion is influenced by a host of factors, it is unclear what these factors are, what impact they have, and whether they are theoretically or practically interesting (4). One uninteresting possibility could be that the lengthy descriptions of the different hospitals that participants were asked to evaluate on six dimensions each reduced participants’ attentiveness to the task, resulting in uninformative responses (5). Without systematically investigating the influence of each deviation from the original studies, it seems like little can be learned from the null findings. Concluding that experiment aversion is neither generalizable nor robust (1) is going far beyond Mazar et al.’s findings.
